# Accidental Phosgene Poisoning: A Case Report and Short Review of Management

**DOI:** 10.7759/cureus.41679

**Published:** 2023-07-11

**Authors:** Sri Hari TY, Dr Sree Sudha TY, KSBS Krishna Sasanka, Konathala Nageswar Rao, Pugazhenthan T

**Affiliations:** 1 Critical Care Medicine, Omni Hospital, Hyderabad, IND; 2 Pharmacology, All India Institute of Medical Sciences, Deoghar, IND; 3 Ear Nose Throat (ENT) & Head Neck Surgery (HNS), All India Institute of Medical Sciences (AIIMS), Deoghar, IND; 4 Emergency Medicine, Omni Hospital, Telangana, IND; 5 Pharmacology and Therapeutics, All India Institute of Medical Sciences (AIIMS), Raipur, IND

**Keywords:** phosgene poisoning, bilateral fluffy alveolar deposits, pneumonitis, triphosgene gas, phosgene gas

## Abstract

Background: Phosgene is a chemical used in the manufacture of plastics and pesticides. Phosgene remains one of the most dangerous of today's high-volume chemicals, as evidenced by the deaths and widespread evacuations caused by its release in industrial accidents. The respiratory system is most severely harmed by exposure to phosgene.

Case Presentation: A 39-year-old male patient arrived feeling short of breath, nauseous, and tachypnoeic after being exposed to triphosgene gas at work. Upon examination, the patient's oxygen saturation (spo2) was 72% without oxygen, 95% on 15 L of oxygen (o2), hemodynamically unstable, and transferred to the intensive care unit (ICU) for additional care. A ventilator was started in non-invasive mode, and antibiotics were administered based on an initial CT scan of the chest that revealed bilateral fluffy alveolar deposits. The same course of treatment was continued on day two. Chest X-ray shadows improved starting on day three. Saturation is 95% after weaning off Niv support and placing 5 L of o2. He was discharged with oral medications once he was hemodynamically stable.

Conclusion: An incidental phosgene poisoning is described in detail here, along with its clinical symptoms and treatment. It is critical to suspect phosgene gas exposure and monitor such patients to save lives.

## Introduction

Phosgene gas is a synthetic, colorless irritant gas heavier than air. It is also known as carbonyl chloride, carbon oxychloride, carbonic acid dichloride, and chloroformyl chloride. When it was employed in chemical warfare during World War I, it first gained a reputation on a global scale. Phosgene is the main agent, which caused about 80% of the 100,000 gas-related casualties [1-3]. The production of dyes, carbamates, acid chlorides, polycarbonates, pharmaceuticals, polyurethanes, isocyanates, and pesticides utilizes phosgene. It also has metallurgical uses. Industrial production across the globe is thought to exceed five billion pounds [4]. The lungs and airways are the most vulnerable to the toxic effects of phosgene. Additionally, olfactory fatigue sets quickly, and the smell of phosgene can be hidden. Phosgene vapor irritates eyes and mucous membranes, but only at levels too high to provide adequate warning (3 ppm). Apnea, eye irritation, and bronchoconstriction are triggered by the inhalation of very high concentrations (0.200 ppm). Acute corpulmonale develops within minutes, and victims pass away as pulmonary blood flow nearly stops. Before more recognizable symptoms of phosgene poisoning have developed, death can happen. Injury develops in three stages after medium exposure doses (150-400 ppm/min). Following the inhalation of concentrations of 0.3 ppm is the reflex phase [5]. Reduced vital capacity, increased arterial partial pressure of carbon dioxide, mild hypoxemia, and mild respiratory acidosis are all effects of shallow, rapid breathing. There may be secondary bradycardia, sinus dysrhythmias, and a drop in systemic blood pressure. This initial phase's intensity varies from person to person, is frequently influenced by emotional factors, and may not be dose-dependent. The reflex phase may not occur after prolonged exposure to concentrations of 3 ppm, even if victims later develop life-threatening lung injury. In the second phase of latency, victims are generally asymptomatic. Although phosgene has immediate biochemical effects, clinical effects can take up to 15 or 30 h in the case of cardiac insufficiency to appear [6]. The inhaled dose has an adverse relationship with the latency phase duration [7-8]. No biological indicators can foretell the beginning of a pulmonary injury. Pulmonary edema associated with phosphonate-induced acute lung injury (ALI) is influenced by concentration and exposure duration (C x t) [9]. Potential remedies for phosphorus-induced pulmonary edema should be pursued. We attempted to provide a succinct overview of the clinical manifestation and treatment of phosgene poisoning.

## Case presentation

**Figure 1:**
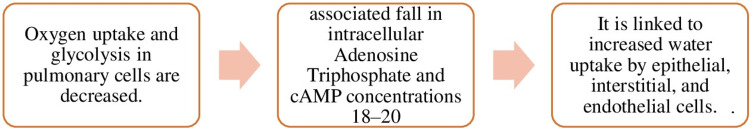
Phosgene exposure - mechanism of action.

**Figure 2:**
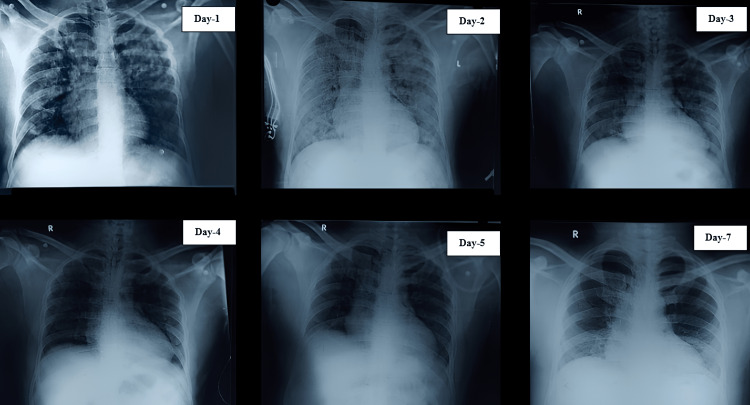
Chest X-ray findings on various days. Bilateral opacities in all lung lobes from Day 1 to Day 4 and is diagnosed as pneumonitis.

**Figure 3:**
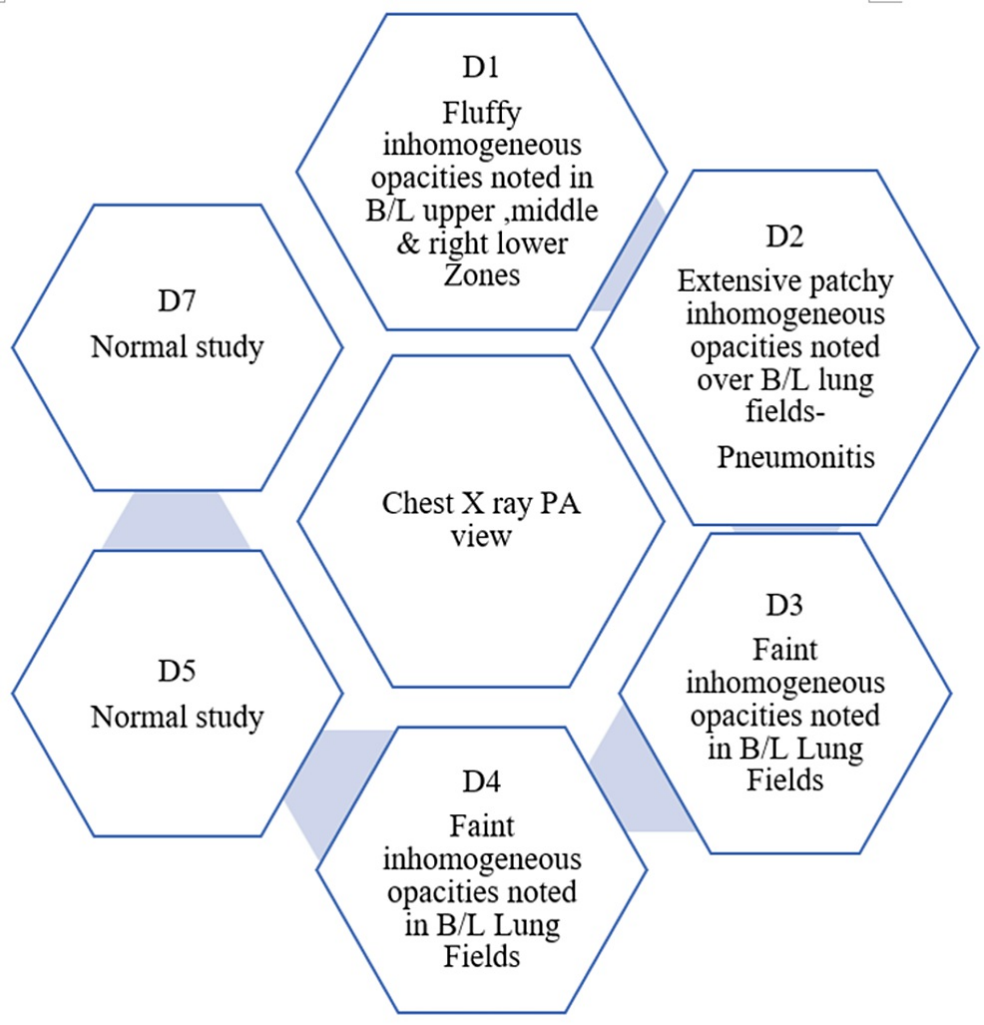
Findings on various days (Day 1 to Day 7). Diagnosed as pneumonitis

A 39-year-old male patient arrived feeling short of breath, nauseous, and tachypnoeic after being exposed to triphosgene gas due to pipeline leakage at work. He is a known diabetic and is being treated. On examination, spo2 was 72% without oxygen, 95% on 15 L oxygen, conscious, temperature 98.6 degrees Fahrenheit, pulse rate 91/min, respiratory rate 34/min, blood pressure 120/80 mmHg, and random blood sugar 139 mg/dL. The patient is having respiratory distress and is transferred to the intensive care unit (ICU) for further evaluation and treatment. On day one, the CT chest showed bilateral fluffy alveolar deposits, a total leucocyte count of 11.7 cells/uL, creatinine 1.1, and HbA1c 6.1. It was started on ventilator non-invasive mode [non-invasive ventilation - a fraction of inspired oxygen 60% (Niv-fio2), positive end-expiratory pressure-6 (PEEP), respiratory rate 20, pressure support - 12], then saturation improved to 100%. On antibiotics injections of 1.5 g of cefoperazone sulbactam, 40 mg of salmeterol, and 10 u s/c of lactus, he was recovering progressively and the 2D echo appeared normal. The same course of treatment was continued on day 2, with ventilator support of NIv-fio2-50%, PEEP-6, respiratory rate-20, and pressure support of 12. Chest X-ray shadows started to improve on day 3 following switching to intermittent Niv. Saturation is 95% after weaning off Niv support and placing 5 L of oxygen. The patient was discharged with oral medications once he was hemodynamically stable. General practitioners advised zomelismet 50/500 twice daily and reviewed it after one week. The mechanism of action for the following phosgene exposure was represented in a flowchart (Figure 1) (see also Figures 2-3).

Management and treatment of phosgene exposure

*Initial management*: Victims need to be removed from the hazardous area. The A, B, C, and Ds of resuscitation must be followed immediately for patients who are unconscious or severely injured. All symptomatic victims with signs of respiratory toxicity, hypoxemia, or severe skin burns should receive advanced life support, such as IV lines and additional oxygen.

*Ventilation using positive airway pressure*: Positive pressure ventilation has been suggested by some authorities as a mechanical defense against phosgene-induced pulmonary edema during the latency phase [9-10]. This method may help to reduce the fluid build-up, stabilize the intra-alveolar surfactant film, and suppress arteriovenous shunts.

*Observation and assessment*: Restrain the victim and closely monitor vital signs and lung auscultation every 30 min. Serial chest roentgenography should be performed starting 2 h after exposure.

*Monitoring and a positive airway pressure*: To support oxygenation and ventilation, endotracheal intubation, mechanical ventilation with positive end expiratory pressure, and high oxygen concentrations are typically needed [11-13]. Continuous positive airway pressure is also advised [14]. Monitoring of pulmonary arterial pressure should be taken into consideration [13].

## Discussion

The amount of phosgene to which a person is exposed, the route of exposure, and the duration of exposure all play a role in phosgene poisoning. Leukotrienes C4, D4, and E4, lipid peroxidation, and oxidized glutathione over time following exposure to phosgene were used in a study to illustrate the mechanism of phosgene poisoning (Figure 1). There was also a significant rise in tracheal pressure, and rate of lung weight gain [14]. When phosgene is inhaled in high concentrations, a series of events occur, including an initial bioprotective phase, a latent period without symptoms, and a terminal phase marked by pulmonary edema. However, a histological examination showed the onset of an edematous swelling with blood plasma increasingly entering the pulmonary interstitium and alveoli. This phase known as the "clinical latent phase," may cause harm to the alveolar type I cells and increase the hematocrit. This phase's duration varies inversely with the inhaled dose [15]. Acute respiratory distress syndrome (ARDS) and acute irritant symptoms can develop within minutes and for up to 72 h because of non-cardiogenic pulmonary edema. Hemoconcentration, hypotension, and hypovolemia may coexist with respiratory symptoms. The onset of these delayed symptoms is usually preceded by a relatively asymptomatic period. It is believed that the length of the latent period is inversely correlated with the severity of the initial symptoms. In some instances, 3-5 weeks after exposure, infectious pneumonitis develops. Although it is also uncommon, death does happen in these situations within 24-48 h of exposure. The majority of patients who recover following phosgene exposure make a full recovery. However, phosgene exposure has been linked to chronic bronchitis and emphysema. Although there is no specific laboratory test for confirming the diagnosis of phosgene gas exposure, various methods should be used to monitor pulmonary status. Initial vital signs and mucous membrane examinations are followed by a chest X-ray, oxygen saturation test, arterial blood gas analysis, and volume status assessment. Similar to our patient, daily chest X-rays have been recorded as well as their findings (Figure 2). On day 1, there were marked opacities bilaterally and it extensively filled all over, confirming pneumonitis by day 2 (Figure 3). The normal study on the chest X-ray was reported by day 6. When pulmonary edema is present, the hila is typically enlarged (4-8 h after exposure) and there are ill-defined patchy infiltrates visible on chest X-rays. To keep a net negative fluid balance, pulmonary edema should be carefully managed. Given that pulmonary edema is not a result of fluid overload, diuretics should be avoided. If required, a central line should be used to monitor the patient's hemodynamics. Antibiotics are administered to our patients by clinical evidence of pneumonia or bronchitis. Early postexposure intervention is necessary for the effective management of phosgene-induced lung injury. This intervention could lower free radical species responsible for lipid peroxidation, balance the glutathione redox state, and stop the release of biological mediators like leukotrienes, which are responsible for increased permeability. Existing bronchospasm may be alleviated by bronchodilators. Numerous medications, including leukotriene antagonists, ibuprofen, colchicine, cyclophosphamide, terbutaline, aminophylline, and isoproterenol, have been shown to have positive effects in animal studies [16]. Because of the irritating effects of phosgene gas, steroids effectively reduce inflammation. Hexamethylenetetramine (HMT) and N-acetylcysteine (NAC) are two particular therapies studied in the past [17-18]. HMT once thought to be a specific antidote, has only been shown to work well when given prophylactically. There is no proof that HMT is advantageous in cases of acute phosgene exposure. Phosgene is thought to be "trapped" by NAC and changed into a less dangerous metabolite. Additionally, it has been suggested that NAC's antioxidant properties contribute to the reduction in direct pulmonary parenchyma toxicity [17]. However, it has not been demonstrated that administering NAC reduces morbidity and mortality in vivo [17-18]. Although theoretically beneficial, nebulized sodium bicarbonate treatment should only be used as a last resort after trying the aforementioned medications.

## Conclusions

Accidental exposure by occupational workers to the highly toxic gas phosgene is possible. This case report details the clinical presentation and treatment of an incident of accidental phosgene poisoning that occurred as a result of a nearby pipeline leak. Given the delay in clinical deterioration seen in some patients who go on to develop adult respiratory distress syndrome, it is imperative to suspect phosgene gas exposure and monitor such patients to save their lives.
